# Circular RNA circTRIM33–12 acts as the sponge of MicroRNA-191 to suppress hepatocellular carcinoma progression

**DOI:** 10.1186/s12943-019-1031-1

**Published:** 2019-06-01

**Authors:** Peng-Fei Zhang, Chuan-Yuan Wei, Xiao-Yong Huang, Rui Peng, Xuan Yang, Jia-Cheng Lu, Chi Zhang, Chao Gao, Jia-Bin Cai, Ping-Ting Gao, Dong-Mei Gao, Guo-Ming Shi, Ai-Wu Ke, Jia Fan

**Affiliations:** 1 Liver Cancer Institute, Zhongshan Hospital, Key Laboratory of Carcinogenesis and Cancer Invasion, Fudan University, Ministry of Education, 180 Fenglin Road, Shanghai, 200032 People’s Republic of China; 2 Cancer Center, Institutes of Biomedical Sciences, Fudan University, Shanghai, 200031 People’s Republic of China

**Keywords:** Hepatocellular carcinoma, Circular RNA, TET1, Histone methylation

## Abstract

**Background:**

Recently, the dysregulation of circular RNA (circRNA) have been shown to have important regulatory roles in cancer development and progression, including hepatocellular carcinoma (HCC). However, the roles of most circRNAs in HCC are still unknown.

**Methods:**

The expression of circular tripartite motif containing 33–12 (circTRIM33–12) in HCC tissues and cell lines was detected by qRT-PCR. The role of circTRIM33–12 in HCC progression was assessed by western blotting, CCK-8, flow cytometry, transwell and a subcutaneous tumor mouse assays both in vitro and in vivo. In vivo circRNA precipitation, RNA immunoprecipitation, luciferase reporter assays were performed to evaluate the interaction between circTRIM33–12 and miR-191.

**Results:**

Here, we found that circTRIM33–12, is downregulated in HCC tissues and cell lines. The downregulation of circTRIM33–12 in HCC was significantly correlated with malignant characteristics and served as an independent risk factor for the overall survival (OS) and recurrence-free survival (RFS) of patients with HCC after surgery. The reduced expression of circTRIM33–12 in HCC cells increases tumor proliferation, migration, invasion and immune evasion. Mechanistically, we demonstrated that circTRIM33–12 upregulated TET1 expression by sponging miR-191, resulting in significantly reduced 5-hydroxymethylcytosine (5hmC) levels in HCC cells.

**Conclusions:**

These results reveal the important role of circTRIM33–12 in the proliferation, migration, invasion and immune evasion abilities of HCC cells and provide a new perspective on circRNAs in HCC progression.

**Electronic supplementary material:**

The online version of this article (10.1186/s12943-019-1031-1) contains supplementary material, which is available to authorized users.

## Background

Hepatocellular carcinoma (HCC) is the third leading cause of cancer-related deaths worldwide [1]. HCC is clinically characterized by its invasiveness, poor prognosis and limited therapeutic opportunities. At present, surgery is the most common form of therapy for HCC, but the characteristics of multifocal development and distant metastases preclude surgical treatment from being curative in most HCC cases [2]; thus, the further understanding of the molecular mechanisms underlying HCC progression is of paramount importance.

Growing evidence has indicated that the dysregulation of circular RNAs (circRNAs) and DNA methylation contribute to tumorigenesis and progression [3–5]. circRNAs are highly conserved and are characterized as covalently closed loop structures with neither a 5′ to 3′ polarity nor a polyadenylated tail; they are RNA transcripts generated by the back-splicing of a single pre-mRNA and have gene-regulatory potential [6, 7]. Recently, studies have revealed that circRNAs were abnormally expressed in several cancer tissues and correlated with disease progression and prognosis [8]. The critical physiological functions of circRNAs include miRNA sponges, protein translation templates and the regulation of parental expression [9–11]. Aberrant circRNA expression also plays an essential role in HCC tumorigenesis and progression [12–14]. The ten eleven translocation (TET) family of methylcytosine dioxygenases (including TET1, TET2, and TET3) participated in converting 5-methylcytosine (5mC) to 5-hydroxymethylcytosine (5hmC), and participated in removing existing DNA methylation tags in cells [15–17]. It has been reported that reduced 5hmC/TET1 expression is associated with the progression of HCC and that increased 5mC expression positively correlates with HCC invasion and recurrence; this suggests that the DNA methylation/demethylation regulated by the TET family of methylcytosine dioxygenases, may participate in the process of HCC progression [18, 19].

Here, we analyzed the expression profiles of tripartite motifs containing 33 (TRIM33)-derived circRNAs in HCC and paired adjacent normal tissues (ANTs) using qRT-PCR. The expression levels of circTRIM33–12 were significantly reduced in HCC tissues compared with the expression levels in ANTs. Kaplan-Meier survival analysis showed that reduced circTRIM33–12 levels correlated with a poorer survival of HCC patients. We examined the functions of circTRIM33–12 in HCC and found that knockdown of circTRIM33–12 promoted cell proliferation, invasion, and immune evasion. In addition, we found that circTRIM33–12 may function as the sponge of oncogenic miR-191 to upregulate TET1 expression and to consequently suppress HCC progression. Therefore, reduced TRIM33–12 expression may serve as a promising biomarker for prognosis prediction and as a potential therapeutic target for HCC patients.

## Methods

### Cell lines and clinical tissues

The seven HCC cell lines used in this study; and the types of tissue samples collected are described in the Additional file 1: Supplementary Materials and Methods.

### Quantitative real-time polymerase chain reaction analysis, western blotting analysis, and immunofluorescence assays

Quantitative real-time polymerase chain reaction (qRT-PCR), immunofluorescence, and western blotting analyses were performed as described in the references and described in the Additional file 1: Supplementary Materials and Methods [20, 21]. The primers and antibodies used in this study are listed in Additional file 2: Table S1 and Additional file 3: Table S2.

### Transfection experiment

The transfection experiments are described in the Additional file 1: Supplementary Materials and Methods. The target sequences of shRNAs are listed in Additional file 4: Table S3.

### Immunohistochemistry

Immunohistochemistry was performed as described in our previous studies [21, 22]. An anti-NKG2D antibody was used to detect the expression of NKG2D. The antibodies used in this study are listed in Additional file 3: Table S2. The intensity of the positive staining was measured as described in the Additional file 1: Supplementary Materials and Methods [20].

### In vivo circRNA precipitation

Biotin-labeled circTRIM33–12 and negative control probes (Additional file 5: Tables S4) were synthesized by Sangon Biotech. The in vivo circRNA immunoprecipitation (circRIP) assay was performed as described in the references and is described in the Additional file 1: Supplementary Materials and Methods [12].

### Knockdown or overexpression of circTRIM33–12

Small hairpin RNAs (shRNAs) of circTRIM33–12 and circTRIM33–12-overexpressing plasmids were synthesized by GenePharma (Shanghai, China), and the shRNA targeting the junction region of the circTRIM33–12 sequence was also synthesized. Huh 7 and SMMC-7721 cells were transfected with circTRIM33–12 shRNA or the overexpressing plasmids using Lipofectamine 2000 (Invitrogen, Carlsbad, CA, USA) according to the manufacturer’s instructions.

### Cell proliferation, migration, and Matrigel invasion assay

Cell proliferation, migration, and Matrigel invasion assays were performed as described in the Additional file 1: Supplementary Materials and Methods [23].

### Flow cytometry assay of the cell cycle

Cells were fixed in 70% ethanol overnight at 4 °C. Then, the fixed cells were resuspended in staining solution (Beyotime, Shanghai) and were incubated for 30 min at 4 °C. Finally, the stained cells were measured by flow cytometry (Beckman Coulter).

### RNA immunoprecipitation (RIP)

RNA immunoprecipitation (RIP) assays were performed using a Magna RIP RNABinding Protein Immunoprecipitation Kit (Millipore) according to the manufacturer’s instructions. The argonaute 2 (AGO2) and IgG antibodies used in this study are listed in Additional file 3: Table S2.

### Luciferase reporter assay

The mutant luciferase reporter vectors were generated using a Mutagenesis Kit (QIAGEN, California, USA) according to the manufacturer’s instructions. Huh 7 cells were seeded into 96-well plates and were cotransfected with a luciferase reporter vector and miR-191 mimics or the negative control using the Lipofectamine 2000 transfection reagent. After 48 h, the firefly and Renilla luciferase activities were quantified with a dual-luciferase reporter assay (Promega, USA).

### In vivo tumor growth and metastasis assays

The in vivo tumor growth and metastasis assays are described in the Additional file 1: Supplementary Materials and Methods.

### Statistical analysis

Statistical analysis was performed with SPSS software (19.0; SPSS, Inc., Chicago, IL) as previously described in reference [22]. In brief, the values are expressed as the mean ± standard deviation (SD). Student’s t test was used for comparisons between groups. The categorical data were analyzed by chi-square or Fisher’s exact tests. Correlation analysis was performed among circTRIM33, TET1, and NKG2D. The cumulative recurrence and survival rates were analyzed using Kaplan-Meier’s method and the log-rank test. Cox’s proportional hazard regression model was used to analyze independent prognostic factors. *P* <  0.05 was considered statistically significant.

## Results

### Dysregulated TRIM33-derived circTRIM33–12 in HCC

**Fig. 1:**
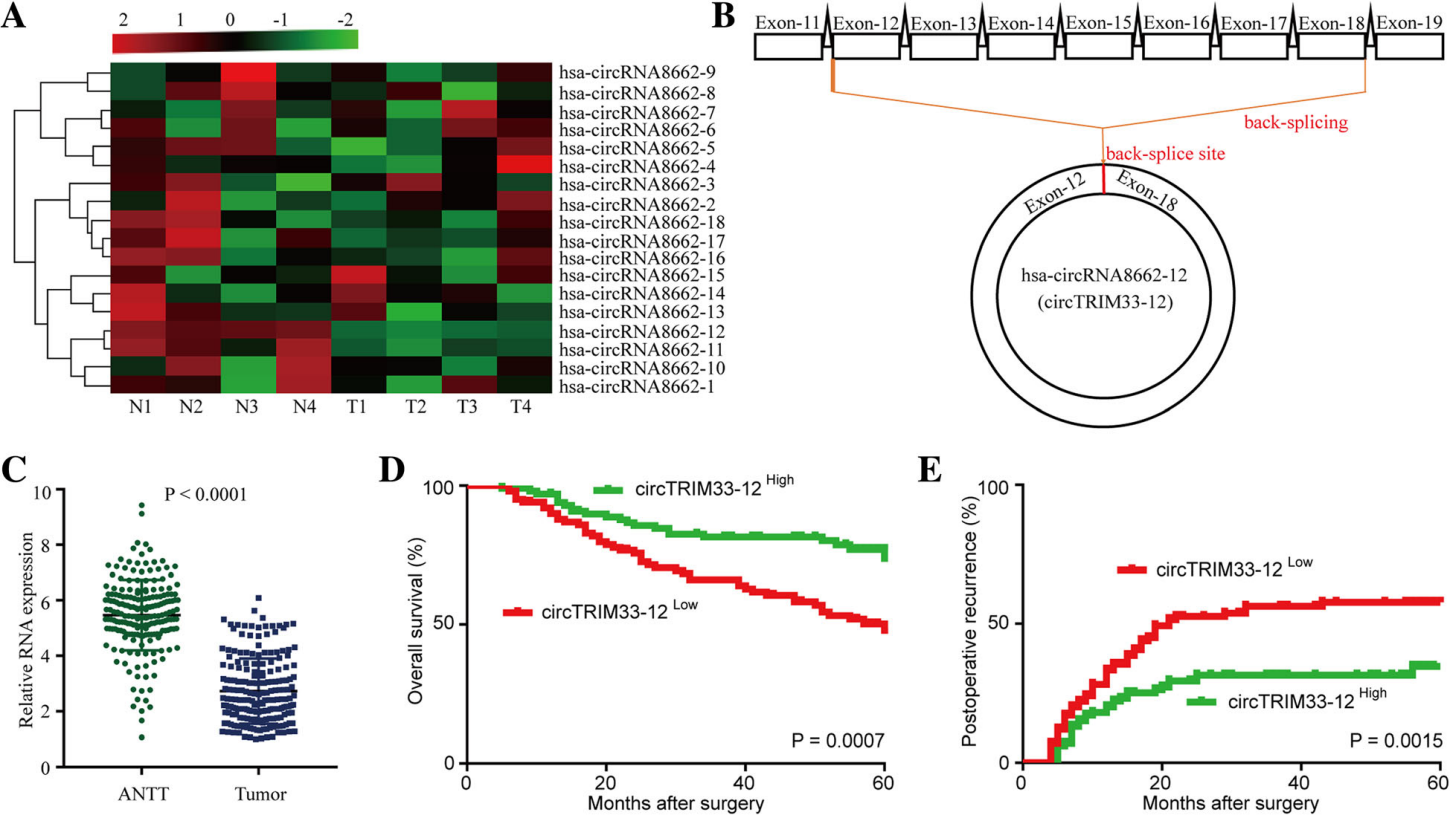
Low circTRIM33–12 expression in HCC tissues and prognostic significance. **a** The heatmap shows circRNAs derived from the TRIM33 gene in HCC tissues compared with those in adjacent nontumor tissues analyzed by qRT-PCR. **b** The scheme illustrating the production of circTRIM33–12. **c** The differential expression of circTRIM33–12 in HCC tissues and adjacent nontumor tissues of 200 patients as indicated. **d** and **e** Prognostic analysis of circTRIM33–12 expression in 200 HCC patients

TRIM33 is a tumor suppressor that can inhibit tumor cell progression and tumorigenesis by different mechanisms in several cancers, including HCC [24–26]. A previous study has reported that the downregulation of TRIM33 in HCC was caused by the hypermethylation of CpG islands in the TRIM33 promoter [24], indicating that circRNA-derived TRIM33 expression might be downregulated in HCC cells. Therefore, we speculated that the possibility of circRNA-derived TRIM33 acts as a tumor suppressor in HCC cells. To investigate TRIM33-derived circRNA expression in HCC tissues, we analyzed four pairs of HCC tissue samples (four HCC tissues and matched adjacent nontumor liver tissues) by using qRT-PCR. Among 18 circRNAs, we found that circTRIM33–12 (hsa_circRNA8662–12) expression was significantly and consistently reduced in four of the HCC tumor tissues compared to that in the matched adjacent nontumor liver tissues (Fig. 1a). Thus, we focused on the expression and role of circTRIM33–12 (circRNA derived from exons 12 and 18 of the TRIM33) for further study (Fig. 1b). To further investigate the prognostic significance of circTRIM33–12 expression in HCC patients, the levels of circTRIM33–12 were quantified in a cohort of 200 HCC and adjacent tissues using qRT-PCR (Fig. 1c). The results showed that circTRIM33–12 was significantly reduced in 58% (116/200) of the HCC tissues examined compared to the levels of circTRIM33–12 in the matched adjacent tissues from the same patient. The median relative expression level of circTRIM33–12 from all 200 HCC tissues was chosen as the cut-off point for separating tumors with the high expression of circTRIM33–12 and the low expression of circTRIM33–12. Next, we investigated the relationship between circTRIM33–12 expression and the clinicopathological characteristics in 200 HCC patients, as listed in Table 1. The results demonstrate that HCC patients with circTRIM33–12^low^ had larger tumor sizes (*P* = 0.022), multiple tumors (*P* = 0.028), encapsulation invasion (*P* = 0.007), elevated alphafetoprotein levels (AFP, *P* = 0.02), and microvascular invasion (*P* = 0.002) compared with those in HCC patients with circTRIM33–12^high^. Kaplan-Meier survival analysis revealed that patients with low circTRIM33–12 expression levels had a poor clinical outcome (Fig. 1d and e). Multivariate analysis identified circTRIM33–12 expression as an independent predictor for overall survival (OS) and postoperative recurrence (Tables 2 and 3). Together, these data suggest that decreased circTRIM33–12 expression in HCC cells is correlated with the poor prognosis of patients and indicate that reduced circTRIM33–12 expression is likely involved in the progression of HCC.

**Table 1:** Correlation between circTRIM33–12 and clinicopathological characteristics in 200 HCCs

Variable	circTRIM33–12		*P* value
	Low	High	
Age (years)			
≤ 50	44	46	1.000
> 50	56	55	
Sex			
Female	13	17	0.553
Male	87	83	
HBsAg			
Negative	21	20	1.000
Positive	79	80	
HCVAb			
Negative	3	4	1.000
Positive	97	96	
Liver cirrhosis			
No	87	83	0.553
Yes	13	17	
Serum AFP, ng/mL			
≤ 20	30	47	0.02
> 20	70	53	
Serum ALT, U/L			
≤ 75	86	86	1.000
> 75	14	14	
Tumor size (diameter, cm)			
≤ 5	49	66	0.022
> 5	51	34	
Encapsulation invasion			
Absent	38	58	0.007
Present	62	42	
Vascular invasion			
No	51	73	0.002
Yes	49	27	
Tumor number			
Single	75	88	0.028
Multiple	25	12	
TNM			
I/ II	73	71	0.875
III/IV	27	29

**Table 2:** Univariate and Multivariate Analyses of Factors Associated with Overall Survival

	OS			
		Multivariate		
Factors	Univariate, P	HR	95% CI	P value
Sex (female vs. male)	0.106			NA
Age (years) (≤50 vs. > 50)	0.118			NA
HBsAg (positive vs. negative)	0.730			NA
HCVAb (positive vs. negative)	0.860			NA
Liver cirrhosis (yes vs. no)	0.598			NA
Serum AFP, ng/mL (≤20 vs. > 20)	0.424			NA
Serum ALT, U/L (≤75 vs. > 75)	0.627			NA
Tumor size (diameter, cm) (> 5 vs ≤ 5)	0.004	1.172	1.066–2.750	0.026
Tumor number (multiple vs. single)	0.175			NA
TNM (III/IV vs I/II.)	0.121			NA
CircTRIM33–12 expression (high vs. low)	0.001	0.504	0.932–1.942	0.007

*OS* overall survival, *NA* not adopted, *AFP* alpha-fetoprotein, *HBsAg* hepatitis B surface antigen, *95%CI* 95% confidence interval, *HR* hazard ratio, Cox proportional hazards regression model

**Table 3:** Univariate and Multivariate Analyses of Factors Associated with Cumulative Recurrence

	Cumulative Recurrence			
		Multivariate		
Factors	Univariate, P	HR	95% CI	*P* value
Sex (female vs. male)	0.161			NA
Age (years) (≤50 vs. > 50)	0.184			NA
HBsAg (positive vs. negative)	0.419			NA
HCVAb (positive vs. negative)	0.708			NA
Liver cirrhosis (yes vs. no)	0.489			NA
Serum AFP, ng/mL (≤20 vs. > 20)	0.182			NA
Serum ALT, U/L (≤75 vs. > 75)	0.622			NA
Tumor size (diameter, cm) (> 5 vs ≤ 5)	< 0.001	2.055	1.335–3.163	0.001
Tumor number (multiple vs. single)	0.131			NA
TNM (III/IV vs I/II.)	0.099			NA
CircTRIM33–12 expression (high vs. low)	0.001	0.534	0.344–0.830	0.005

*NA* not adopted, *AFP* alpha-fetoprotein, *HBsAg* hepatitis B surface antigen, *95%CI* 95% confidence interval, *BCLC* Barcelona-Clinic Liver Cancer, *HR* hazard ratio, Cox proportional hazards regression model

### circTRIM33–12 inhibits the proliferation and invasion of HCC cells

Next, the biological functions of circTRIM33–12 in HCC progression were investigated. To choose the most suitable HCC cell lines used for the knockdown or overexpression of circTRIM33–12, we detected the expression of circTRIM33–12 in seven HCC cell lines. SMMC-7721 and HCCLM3 cells showed a lower expression of circTRIM33–12, and Huh 7, HepG2, and MHCC97L cells showed a higher expression of circTRIM33–12 (Additional file 6: Figure S1a). Therefore, Huh 7 and MHCC97L cells were used for the knockdown of circTRIM33–12; SMMC-7721 and HCCLM3 cells were used for the overexpression of circTRIM33–12. Using small interfering RNAs (siRNAs) targeting the back-splicing sequence, we effectively knocked down circTRIM33–12 in Huh 7 and MHCC97L cells (Additional file 6: Figure S1b). To convincingly show that circTRIM33–12 siRNA had no effects on other TRIM33 splicing products, qRT-PCR was performed to detect the TRIM33 mRNA expression. The expression of TRIM33 mRNA showed no significant change after the transfection of circTRIM33–12 siRNA, confirming the specificity of circTRIM33–12 silencing (Additional file 6: Figure S1c). Furthermore, using the plasmid vector, we succeeded in overexpressing circTRIM33–12 in SMMC-7721 and HCCLM3 cells and further obtained stable circTRIM33–12-overexpressing SMMC-7721 and HCCLM3 clones which showed approximately four fold overexpression (Additional file 6: Figure S1d).

**Fig. 2:**
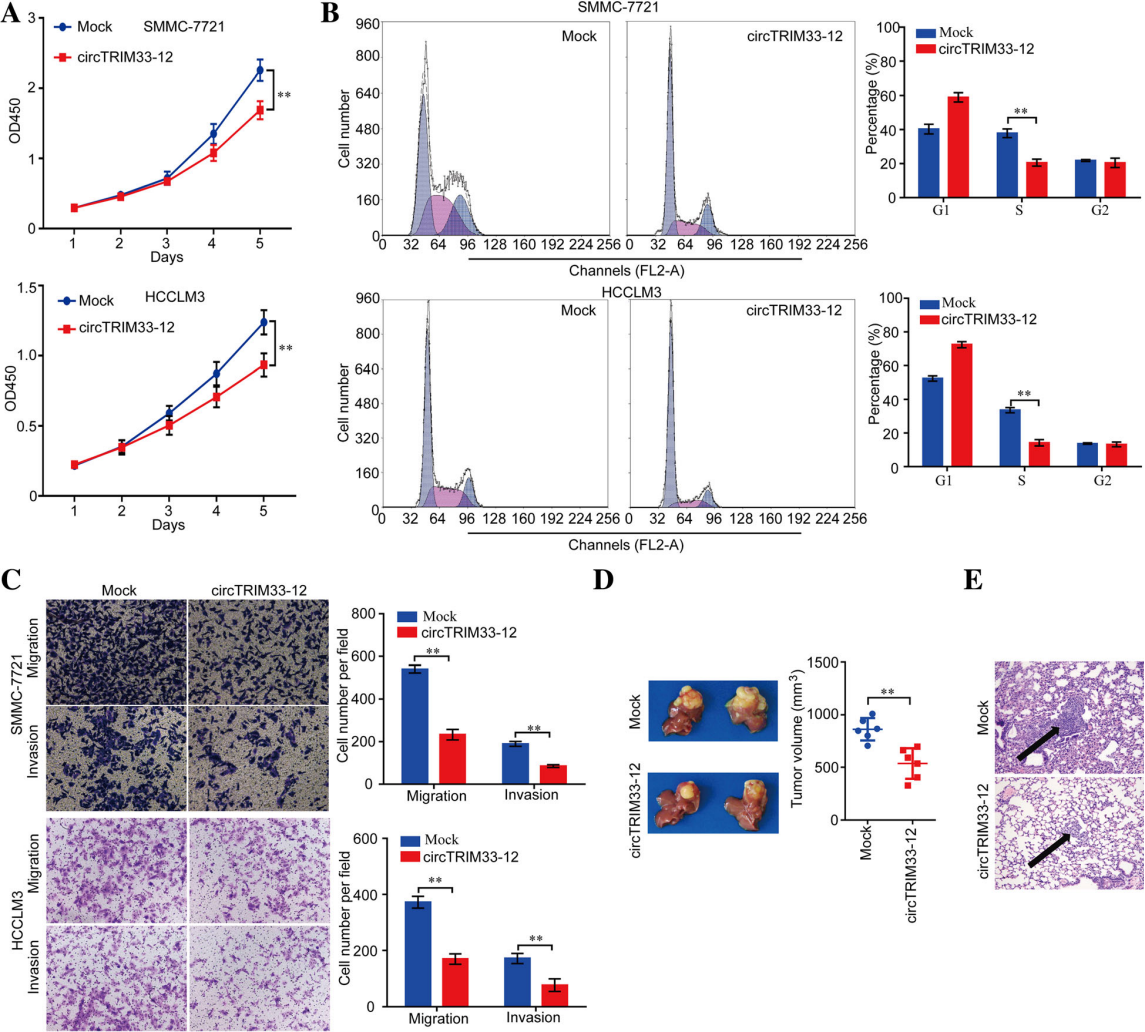
High circTRIM33–12 expression inhibited the progression of HCC cells in vitro and in vivo. **a** Cell proliferation in HCC cells with the forced expression of circTRIM33–12 was assessed by a CCK-8 assay. **b** The cell cycle in HCC cells with the forced expression of circTRIM33–12 was detected by FCM. **c** The migration and invasion abilities of HCC cells with the forced expression of circTRIM33–12 was evaluated via a transwell assay. **d** Tumor growth in HCC cells with the forced expression of circTRIM33–12 was investigated by nude mice xenograft tumor models. **e** Serial sections from mouse lungs showed the metastatic ability of cancer cells expressing different levels of circTRIM33–12. The data are represented as the mean ± SD, *n* = 3. **P < 0.01

The CCK-8 assay, cell cycle assay, and transwell migration and invasion assay revealed a significant inhibition in the growth and metastasis of SMMC-7721 and HCCLM3 cells after overexpressing circTRIM33–12 compared with the growth and metastasis of the mock control (Fig. 2a-c). Moreover, the knockdown of circTRIM33–12 expression promoted the growth and metastasis of Huh 7 and MHCC97L cells (Additional file 6: Figure S2a-c). To further explore the effects of circTRIM33–12 in vivo, circTRIM 33–12-overexpressing SMMC-7721 cells and mock control cells were applied to establish orthotopic implanted intrahepatic HCC models. The size of the tumors was smaller in the circTRIM33–12-overexpressing group compared with the size of the tumors in the mock control after in situ growth for 4 weeks in SMMC-7721 xenografts (Fig. 2d). Consistent with these expectations, the metastatic nodules in the pulmonary tissues were reduced in the circTRIM33–12-overexpressing group compared with those in the mock control group in SMMC-7721 xenografts (Fig. 2e).

### circTRIM33–12 may function as a sponge for miR-191

**Fig. 3:**
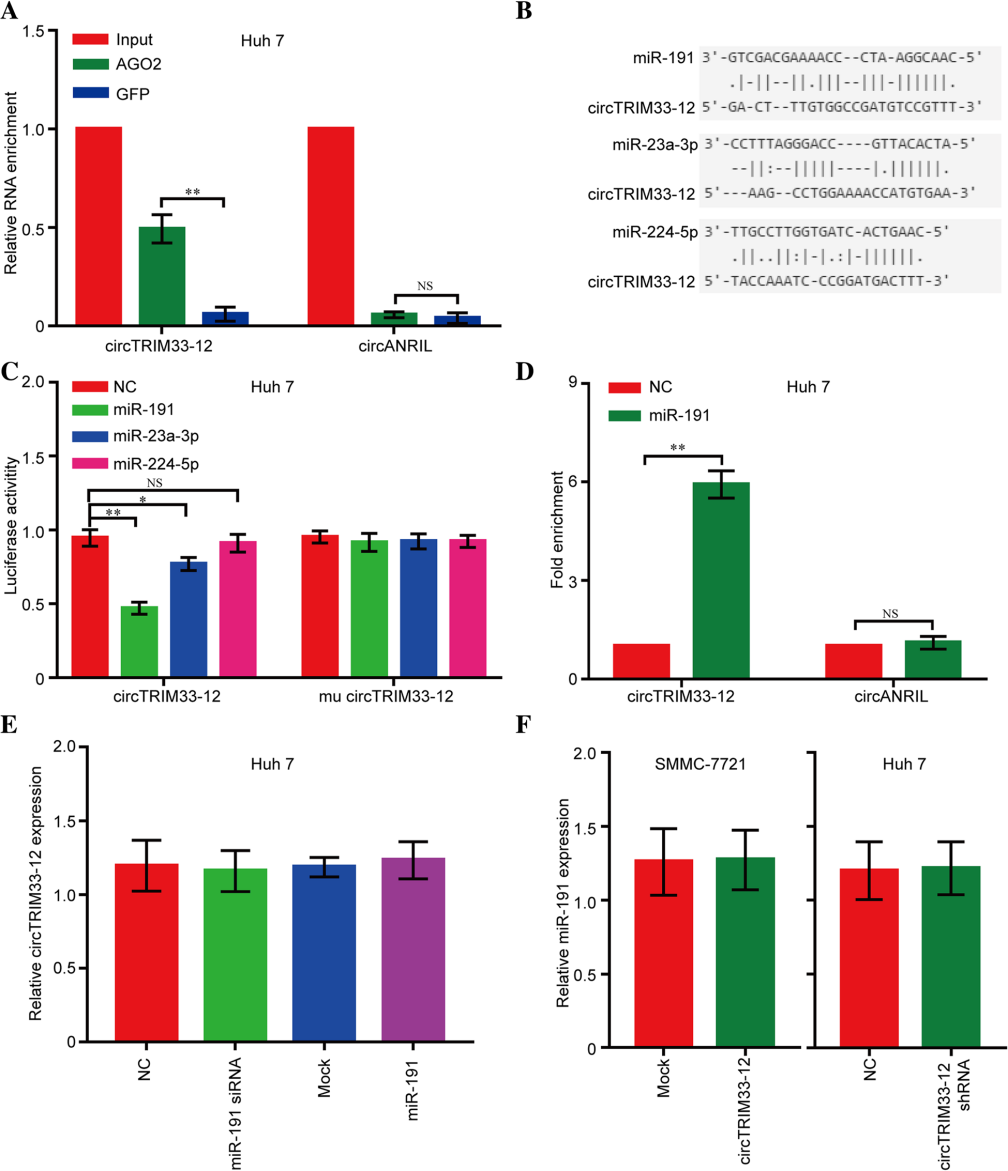
circTRIM33–12 binds miR-191 in HCC cells. **a** RIP experiments were performed using an antibody against AGO2 on extracts from Huh 7 cells. **b** A schematic drawing showing the putative binding sites of miR-23a-3p, miR-191, and miR-224-5p with respect to circTRIM33–12. **c** The luciferase activity of luc-circTRIM33–12 or mutant luc-circTRIM33–12 in Huh 7 cells after cotransfection with miR-23a-3p, miR-191, or miR-224-5p. **d** circRIP was performed in Huh 7 cells using biotin-labeled miR-191 mimics and a negative control (NC). The enrichment of circTRIM33–12 was detected by qRT-PCR and was normalized to that of the NC. **e** The expression of circTRIM33–12 in Huh 7 cells after transfection with miR-191 or miR-191 siRNA. **f** The expression of circ miR-191 in SMMC-7721 and Huh 7 cells after transfection with circTRIM33–12 or circTRIM33–12 shRNA. The data are represented as the mean ± SD, *n* = 3. *P < 0.05; **P < 0.01; NS, no significant

Given that circRNAs have been shown to act as miRNA sponges in cancer cells, we explored whether circTRIM33–12 can bind to certain miRNAs in the progression of HCC. First, we conducted RIP with an antibody against AGO2 in Huh 7 cells. The results showed that circTRIM33–12, but not cANRIL (a circular RNA reported not to bind to AGO2), was significantly enriched by the AGO2 antibody (Fig. 3a), suggesting that circTRIM33–12 may act as a binding platform for AGO2 and miRNAs. Next, we used the StarBase v2.0 target prediction tool to find 11 potential miRNAs that could bind to circTRIM33–12. According to recent studies, three these of miRNAs act as oncogenes, including miR-23a-3p, miR-191, and miR-224-5p. To further identify the miRNAs miR-23a-3p, miR-191 and miR-224-5p that bind to circTRIM33–12, we constructed a circTRIM33–12 expression plasmid vector containing a luciferase gene and transfected the plasmid vector into Huh 7 cells. After transfection, we observed that 3 miRNAs, particularly miR-191, showed reduced levels of luciferase activity (Fig. 3b and c). Furthermore, the results of a pull-down assay using biotin-labeled miR-191 mimics showed obvious enrichment of circTRIM33–12 compared with that of the negative control (NC) (Fig. 3d). However, circTRIM33–12 did not show significant changes after increased or reduced miR-191 mimic expression in HCC Huh 7 cells (Fig. 3e), and neither miR-191 showed significant changes after the forced expression or knockdown of circTRIM33–12 expression in HCC SMMC-7721 or Huh 7 cells (Fig. 3f). These findings suggest that circTRIM33–12 and miR-191 may not be degraded by each other. All these experiments suggest that circTRIM33–12 may function as a sponge for miR-191.

### circTRIM33–12 upregulates TET1 expression via sponging miR-191

**Fig. 4:**
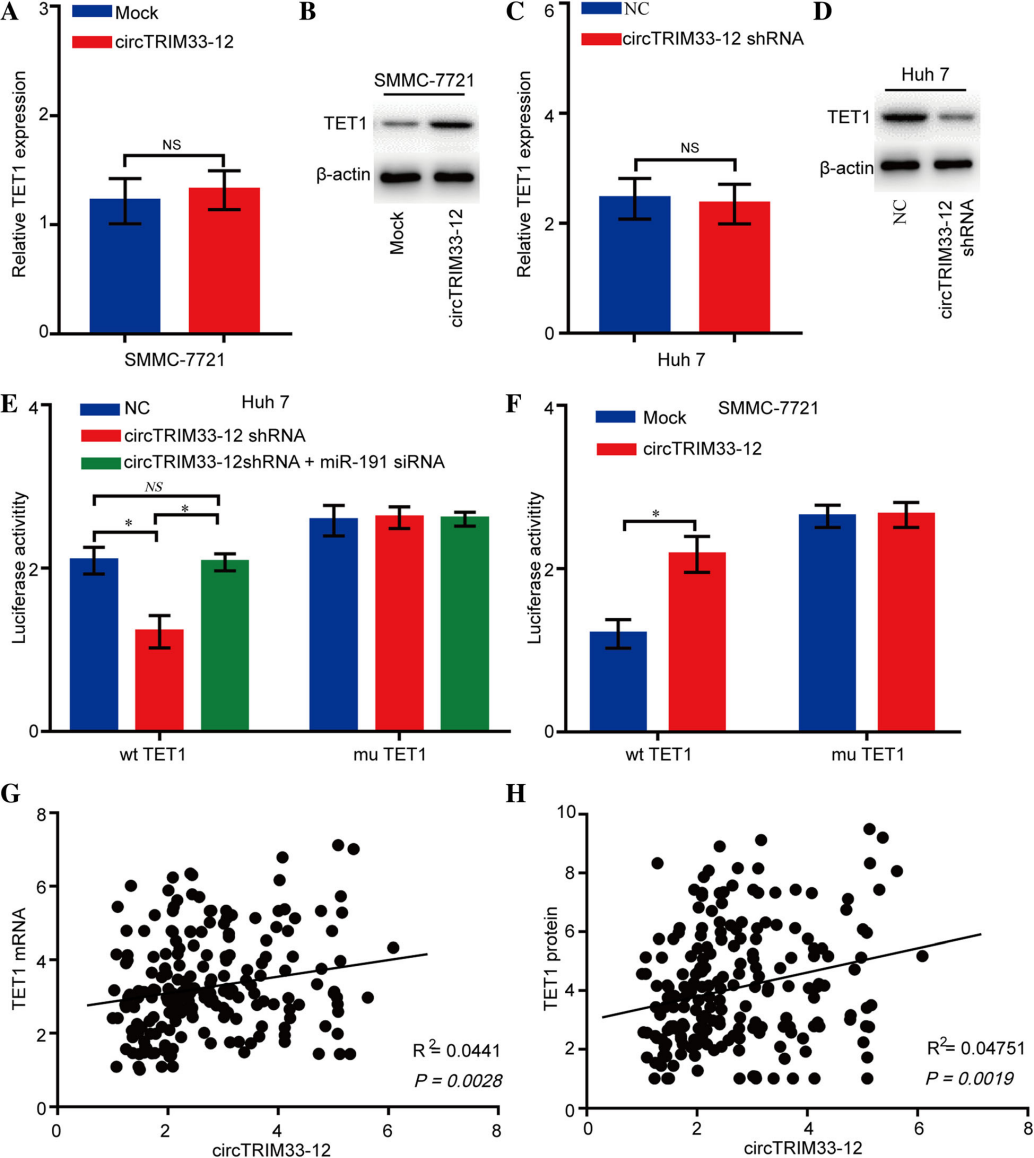
circTRIM33–12 upregulates TET1 expression in HCC cells. **a-d** The relative mRNA and protein levels of TET1 were detected in SMMC-7721 and Huh 7 cells after transfection with circTRIM33–12, circTRIM33–12 shRNA, or the control using qRT-PCR and western blotting, respectively. **e** The luciferase activity of luc-TET1 or mutant luc-TET1 in Huh 7 cells after cotransfection with circTRIM33–12 shRNA or circTRIM33–12 and miR-191 siRNA. **f** The luciferase activity of luc-TET1 or mutant luc-TET1 in SMMC-7721 cells after cotransfection with circTRIM33–12 or the mock control. **g** and **h** The expression of TET1 in 200 cases of HCC tissues was detected by qRT-PCR and IHC. A positive correlation between circTRIM33–12 and TET1 was observed in tumor tissues at the protein (R2 = 0.0441; *P* = 0.0028) and mRNA (R2 = 0.04751; *P* = 0.0019) levels. The data are represented as the mean ± SD, *n* = 3. *P < 0.05; NS, no significant

Previous studies have reported that several genes are directly targeted by miR-191 in human cancers including TET1, TIMP3, SATB1, and DIECR1 [27–29]. However, we did not detect significant changes in the expression levels of these mRNAs in Huh 7 cells transfected with a miR-191 mimic or in SMMC-7721 cells transfected with a miR-191 siRNA. Interestingly, we also did not detect significant changes in the expression levels of these proteins in miR-191-overexpressing/knockdown cells except for the expression of the TET1 protein (Additional file 6: Figure S3a-d). To verify whether the 3′ UTR of TET1 mRNA was a target of miR-191 in HCC cells, a luciferase reporter gene assay was used. The wild-type (wt) 3′ UTR sequence or mutant (mu) 3′ UTR sequence of TET1 was cloned into a luciferase reporter vector. The luciferase activity was significantly inhibited by the miR-191 mimics in wt 3′ UTR sequence-transfected Huh 7 cells. Conversely, the luciferase activity was not inhibited by the miR-191 mimics in mu 3′ UTR sequence-transfected Huh 7 cells (Additional file 6: Figure S3e and f). These findings indicate a direct interaction between miR-191 and TET1 mRNA in HCC cells. To further confirm the effects of circTRIM33–12 on TET1 expression, SMMC-7721 cells were transfected with the circTRIM33–12 plasmid, and the TET1 mRNA and protein levels were detected using qRT-PCR and western blotting. The results showed that the ectopic expression of circTRIM33–12 significantly increased the TET1 protein levels but not mRNA levels in SMMC-7721 cells (Fig. 4a and b). Conversely, the knockdown of circTRIM33–12 expression significantly reduced the TET1 protein levels but not mRNA levels in Huh 7 cells (Fig. 4c and d). A luciferase activity assay demonstrated that the knockdown of circTRIM33–12 could effectively reduce the luciferase activity of the wt but not the mu 3′ UTR of TET1, and the inhibition of luciferase activity caused by circTRIM33–12 knockdown could be restored by miR-191 inhibition in Huh 7 cells (Fig. 4e and f). Moreover, the forced expression of circTRIM33–12 could effectively increase the luciferase activity of the wt but not the mu TET1 3′ UTR in SMMC-7721 cells (Fig. 4f). Finally, we determined the expression of TET1 in 200 HCC patient tissues using qRT-PCR and IHC. A positive relationship between circTRIM33–12 and TET1 was found in HCC patients (Fig. 4g and h).

### circTRIM33–12 and TET1 are responsible for regulating 5hmC expression

**Fig. 5:**
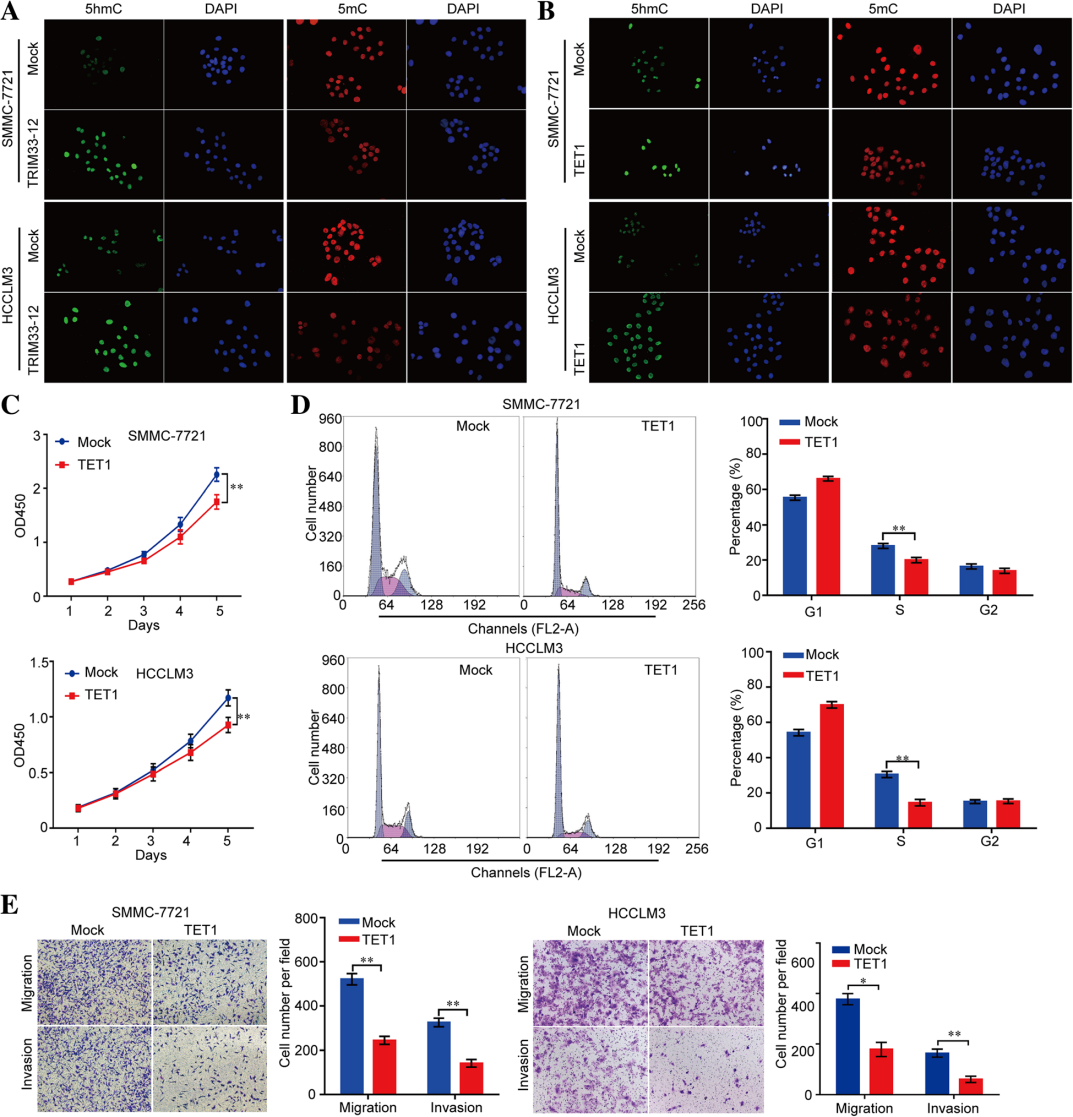
High TET1 expression inhibited the progression of HCC cells in vitro. **a** The expression of 5hmC and 5mC was detected in SMMC-7721 cells after transfection with circTRIM33–12 or the control using IF. **b** The expression of 5hmC and 5mC was detected in SMMC-7721 cells after transfection with TET1 or the control using IF. **c** Cell proliferation in HCC cells with the forced expression of TET1 was assessed by a CCK-8 assay. **d** The cell cycle in HCC cells with the forced expression of TET1 was detected by FCM. **e** The migration and invasion abilities in HCC cells with the forced expression of TET1 were evaluated via a transwell assay. The data are represented as the mean ± SD, *n* = 3. *P < 0.05; **P < 0.01

To test whether the altered circTRIM33–12 or TET1 expression could regulate the DNA methylation levels in HCC cells, circTRIM33–12 expression in HCC cells was modified by shRNA interference and cDNA transfection (Additional file 6: Figure S4a-c). We first compared the 5mC and 5hmC levels in mock- and circTRIM33–12- or TET1-overexpressing SMMC-7721 and HCCLM3 cells. We found that the levels of 5hmC were significantly increased in the circTRIM33–12- or TET1-overexpressing cells, as shown by the IF assay (Fig. 5a and b). Conversely, the levels of 5hmC were significantly reduced in circTRIM33–12 or TET1-knockdown Huh 7 and MHCC97L cells as shown by the IF assay (Additional file 6: Figure S5a-b). Interestingly, growth curves demonstrated that TET1 significantly inhibited cell proliferation compared with the cell proliferation of the mock groups (Fig. 5c). Moreover, in the cell cycle assay, TET1 reduced the cell number in the S phase compared with those in the mock groups (Fig. 5d). In vitro migration and invasion assays were performed to explore whether TET1 inhibited the migration and invasive capacity of tumor cells. In addition to circTRIM33–12, TET1 significantly inhibited SMMC-7721 cell migration and invasion compared with those of the mock group (Fig. 5e). Moreover, the capacity of proliferation, migration and invasion were significantly inhibited in TET1 knockdown Huh 7 and MHCC97L cells as shown by the CCK-8, cell cycle, and transwell assays (Additional file 6: Figure S5c-e). These data convincingly demonstrate that circTRIM33–12 suppresses HCC progression as a sponge of miR-191 to inhibit the miR-191 oncogenic effects through the circTRIM33–12/miR-191/TET1 axis.

### TET1 negatively regulates oncogene expression and is related to immune evasion

**Fig. 6:**
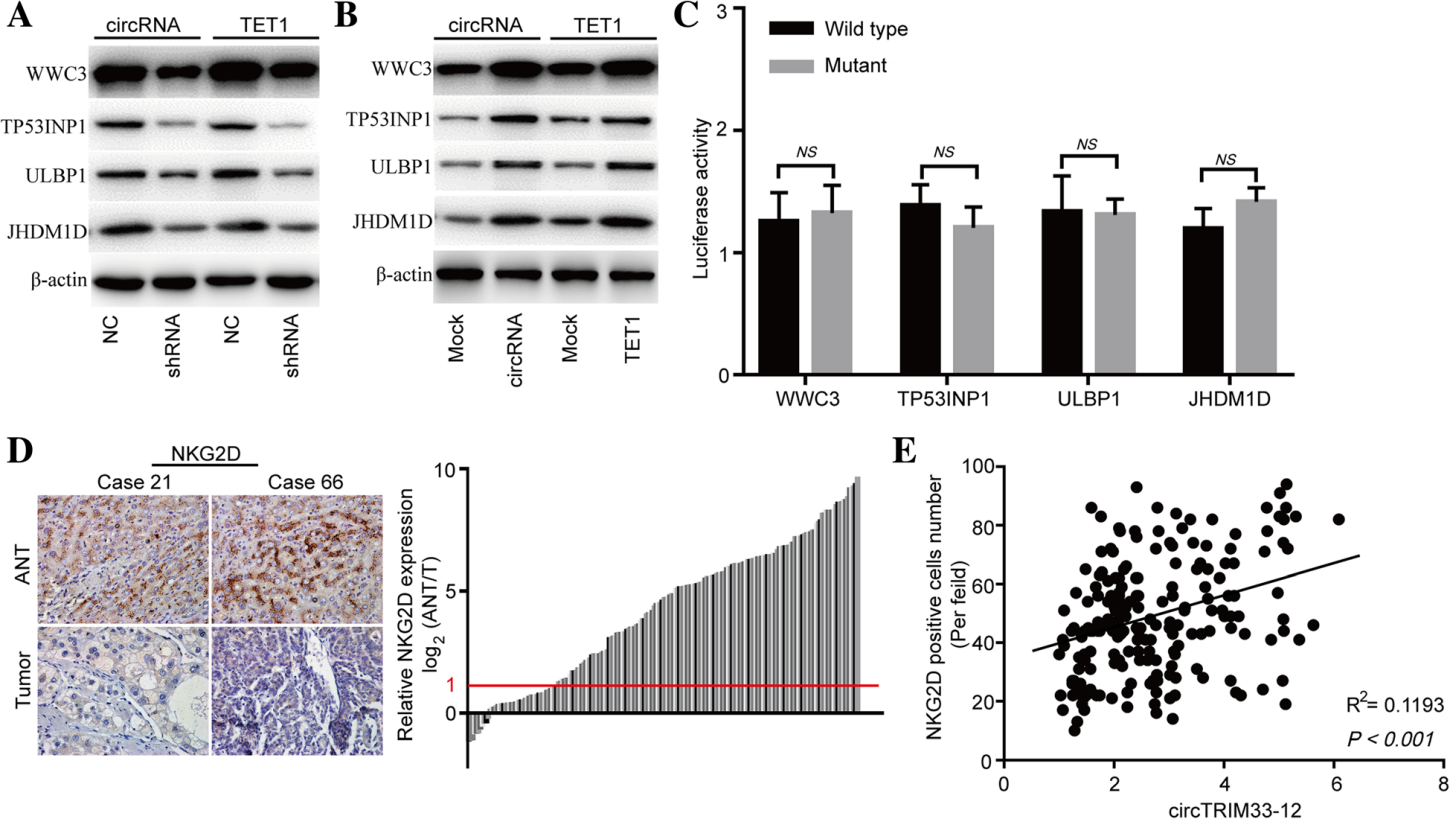
circTRIM33–12 and TET1 regulate the expression of several same genes in HCC cells. **a** The protein levels of WWC3, TP53INP1, ULBP1 and JHDM1D were detected in SMMC-7721 cells after transfection with circTRIM33–12, TET1, or the control using western blotting. **b** The protein levels of WWC3, TP53INP1, ULBP1 and JHDM1D were detected in Huh 7 cells after transfection with circTRIM33–12 shRNA, TET1 shRNA, or the control using western blotting. **c** The luciferase activity of luc-WWC3/TP53INP1/ULBP1/JHDM1D or mutant luc-WWC3/TP53INP1/ULBP1/JHDM1D in Huh 7 cells after cotransfection with miR-191. **d** Representative HCC cases in the tissue microarray were analyzed by immunohistochemical staining for NKG2D. **e** A positive correlation between circTRIM33–12 and the number of NKG2D-positive cells was observed in HCC tissues (R^2^ = 0.1193; *P* < 0.001). The data are represented as the mean ± SD, *n* = 3. NS, no significant

To further gain a deeper mechanistic understanding of the circTRIM33–12/ miR-191/ TET1 axis, potential transcriptomic regulation by TET1 was explored in TET1 knockdown and negative control Huh 7 cells using RNA sequencing analysis. 5mC is an important epigenetic marker associated with transcription repression. We next sought to investigate whether TET1 knockdown transcriptionally inhibits tumor suppressor gene expression in HCC cells. The RNA-seq results showed that TET1 knockdown downregulated several tumor suppressor genes that participated in tumor progression, such as WWC family member 3 (WWC3), tumor protein p53 inducible nuclear protein 1 (TP53INP1), UL16 binding protein 1 (ULBP1) and lysine demethylase 7A (JHDM1D) (Additional file 7: Table S5). To investigate whether circTRIM33–12 exerts its biological functions through the miR-191/TET1 axis, we examined the expression of WWC3, TP53INP1, ULBP1 and JHDM1D in HCC cells with circTRIM33–12 or TET1 knockdown and overexpression. Both the silencing of circTRIM33–12 or TET1 significantly reduced the mRNA and protein expression levels of WWC3, TP53INP1, ULBP1 and JHDM1D in HCC Huh 7 cells (Fig. 6a; Additional file 6: Figure S6a). In addition, the overexpression of circTRIM33–12 or TET1 could increase the mRNA and protein expression levels of WWC3, TP53INP1, ULBP1 and JHDM1D in HCC SMMC-7721 cells (Fig. 6b; Additional file 6: Figure S6b). To verify whether the 3′ UTR of the WWC3, TP53INP1, ULBP1 and JHDM1D mRNAs were targets of miR-191 in HCC cells, a luciferase reporter gene assay was used. The 3′ UTR sequences of WWC3, TP53INP1, ULBP1 and JHDM1D were cloned into a luciferase reporter vector. The luciferase activity was not inhibited by the miR-191 mimics in 3′ UTR sequence-transfected Huh 7 cells (Fig. 6c). These findings indicate that WWC3, TP53INP1, ULBP1 and JHDM1D mRNAs are not targets of miR-191 in HCC cells.

The activating receptor natural-killer group 2 member D (NKG2D) and its ligands play vital roles in NK cell-, γδ + T cell- and CD8+ T cell-mediated immune responses to cancers [30, 31]. NKG2D recognizes eight different ligands in humans, including ULBP1 [32]. One of the important mechanisms that prevents cancer progression is immune surveillance against cancer cells, in which NK cells and CD8+ T cells play a crucial role [33, 34]. To further explore the relationship between circTRIM33–12 and immune evasion, we examined the expression of NKG2D in tissues from 200 cases of HCC and the matched nontumor tissues. The number of NKG2D-positive cells in HCC tissues were significantly reduced compared with that in the adjacent nontumor tissues (156/200; 2 fold) (Fig. 6d). A scatter plot analysis revealed a positive correlation between circTRIM33–12 expression and NKG2D-positive cell number in HCC tissues (R^2^ = 0.1193; *P* <  0.001; Fig. 6e). These results suggest that circTRIM33–12 may exert its antitumor effects by protecting TET1 via sponging miR-191.

### Silencing of miR-191 reversed the knockdown of circTRIM33–12 and induced the progression of HCC cells

**Fig. 7:**
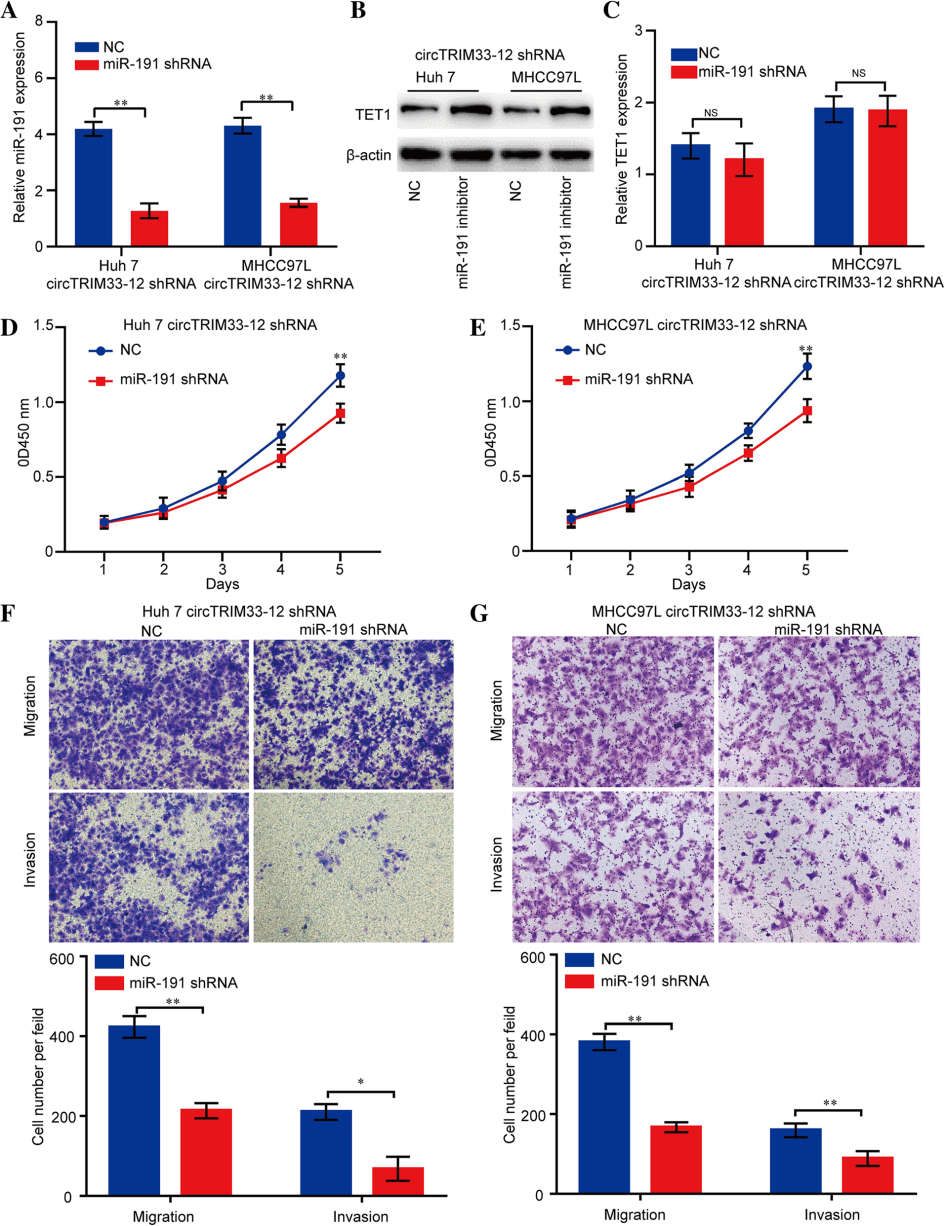
Silencing of miR-191 impairs the cell proliferation, migration and invasion abilities of HCC cells with reduced circTRIM33–12 expression. **a** miR-191 expression in HCC cells was modified by shRNA interference. **b**, **c** Reduced miR-191 expression enhanced TET1 protein expression but not TET1 mRNA in HCC cells. **d**, **e** The cell proliferation in HCC cells with the reduced expression of miR-191 was assessed by a CCK-8 assay. **f**, **g** The migration and invasion abilities in HCC cells with the reduced expression of miR-191 were evaluated via a transwell assay. The data are represented as the mean ± SD, *n* = 3. *P < 0.05; **P < 0.01; NS, no significant

We next explored whether the silencing of miR-191 affected circTRIM33–12 knockdown HCC cell proliferation, migration, and invasion. The silencing efficiency was detected by qRT-PCR (Fig. 7 a). As expected, the silencing of miR-191 upregulated TET1 protein expression but not mRNA expression in both Huh 7 and MHCC97L cell lines with circTRIM33–12 knockdown (Fig. 7 b and c). Furthermore, in the CCK-8 and transwell assays, the silencing of miR-191 significantly inhibited the proliferation, migration, and invasion abilities of Huh 7 and MHCC97L cell lines with reduced levels of circTRIM33–12 expression (Fig. 7d-g). Collectively, these results suggest that circTRIM33–12 suppresses HCC cell progression through the circTRIM33–12/miR-191/TET1 axis.

## Discussion

In this study, we first discovered that circTRIM33–12 is frequently downregulated in HCC, and its expression significantly correlated with better clinicopathologic characteristics. Second, our data showed that the low expression of circTRIM33–12 correlated with poor patient prognosis, indicating its applicability as a promising prognostic biomarker in HCC. Third, we demonstrated that the overexpression of circTRIM33–12 reversed the oncogenic function of miR-191 and thus inhibited the progression of HCC. Fourth, we revealed that circTRIM33–12 acts as a ceRNA and regulates TET1-induced DNA demethylation by competing for miR-191. Considering the vital role of TET1-induced DNA demethylation in the progression of cancers, our results unraveled the therapeutic importance of circTRIM33–12 in HCC for the first time.

Currently, the roles of circRNAs in carcinogenesis and cancer progression have attracted much attention; however, the biological functions and molecular mechanisms for circRNA function in these pathological processes have not been elucidated clearly. Recently, several studies have discovered that circRNA works as a miRNA sponge to protect mRNAs from miRNA attack [14, 35]. For example, circMTO1 was reported to be a tumor-related molecule in HCC cells that functions by absorbing miR-9 [14]. Circular RNA cSMARCA5 functions as a sponge of miR-17-3p and miR-181b-5p to suppress the proliferation and migration of HCC cells [12]. circNT5E participates in glioblastoma tumorigenesis by interacting with miR-422a [35]. To investigate the expression and function of circRNAs derived from TRIM33 in HCC pathogenesis, we performed qRT-PCR to detect the expression levels of TRIM33-related circRNAs in 4 pairs of HCC and adjacent nontumor tissues. The results showed that the expression of circTRIM33–12 was reduced in all 4 HCC tissues. Herein, circTRIM33–12 has been shown to target miR-191 using bioinformatics tools. Intriguingly, the ectopic expression of miR-191 reduced the luciferase activity of the wt circTRIM33–12 reporter. However, there was no significant difference in circTRIM33–12 expression upon forced miR-191 expression. Furthermore, endogenous circTRIM33–12 and miR-191 were pulled down by a special AGO2 antibody. Taken together, all of the data suggest that miR-191 recognizes and binds to circTRIM33–12 without promoting the degradation of circTRIM33–12. miR-191 is an oncogene and is involved in cancer progression, including HCC [36, 37]. Because forced miR-191 expression promotes HCC cell proliferation, migration and invasion [36, 38], we reasoned that miR-191 may participate in the regulation of the circTRIM33–12-induced inhibition of proliferation, migration and invasion in HCC cells. TET1 is a methylcytosine dioxygenase that participates in epigenetic regulation [39]. TET1 protein can lead to DNA demethylation by oxidizing 5mC to 5hmC, 5-formylcytosine (5fC), and 5-carboxylcytosine (5caC) [40]. A previous study provided evidence that TET1 exerts its anti-tumor functions in cancer cells by several mechanisms, including suppressing Wnt/β-catenin signaling via the demethylation of Wnt antagonists and promoting tumor suppressor gene expression by demethylating a CpG site within the tumor suppressor gene promoter [41–43]. However, the molecular mechanism of the induction of TET1 and its role in tumor progression are still obscure. Here, we found that circTRIM33–12 abolishes the suppression of TET1 mediated by miR-191, and the knockdown of TET1 contributes to the promotion of proliferation, migration and invasion. Therefore, we assumed that circTRIM33–12 could upregulate TET1 expression by competitively sponging miR-191, thereby inhibiting HCC progression. In addition, circTRIM33–12 facilitated tumor suppressor gene expression in HCC cells, such as WWC3, TP53INP1, ULBP1 and JHDM1D, via sponging the miR-191-induced upregulation of TET1 in HCC cells. Notably, the combination of circTRIM33–12 and miR-191/TET1 could be a potential prognostic biomarker panel of HCC. All of the above data suggest that the circTRIM33–12/miR-191/TET1 axis plays a critical role in HCC.

## Conclusions

In summary, our findings report that circTRIM33–12 expression is significantly reduced in HCC tissues and that low levels of circTRIM33–12 expression correlate with a poor prognosis for HCC patients. Functionally and mechanistically, circTRIM33–12 inhibits HCC proliferation, metastasis and immune evasion by sponging miR-191 and upregulating TET1 expression, indicating its tumor suppressive role in HCC progression. Furthermore, the in vivo experiments and clinical characteristics of circTRIM33–12 indicate its potential in HCC-targeted therapy. Taken together, our data offer a novel therapeutic target, circTRIM33–12, to broaden the treatment options for HCC.

## Additional files

Additional file 1: **Supplementary Materials and Methods (DOC 34 kb)**

Additional file 2: **Table S1**. Sequence of primers for qRT-PCR. (DOCX 17 kb)

Additional file 3: **Table S2**. Antibody for western blotting, immunofluorescence, RIP, and immunohistochemistry. (DOCX 17 kb)

Additional file 4: **Table S3.** Target sequences of TET1 shRNA. (DOCX 15 kb)

Additional file 5: **Table S4.** circTRIM33–12 circRIP probe sequence. (DOCX 16 kb)

Additional file 6: **Figure S1.** circTRIM33–12 expression in HCC cells. **Figure S2.** circTRIM33–12 regulated the progression of HCC cells in vitro. **Figure S3.** TET1 binds to miR-191 in HCC cells. **Figure S4.** Forced or reduced TET1 expression in HCC cells. **Figure S5.** TET1 regulated the progression of HCC cells in vitro. **Figure S6.** circTRIM33–12 and TET1 regulate the expression of several same genes in HCC cells. (DOCX 3576 kb)

Additional file 7: **Table S5.** The significant reduced genes in TET1 knockdown cells. (XLSX 12 kb)

## Abbreviations

5caC: 5-carboxylcytosine
5fC: 5-formylcytosine
5hmC: hydroxymethylcytosine
5mC: 5-methylcytosine
AFP: Alphafetoprotein
AGO2: Argonaute 2
ANT: Adjacent normal tissue
circRNA: Circular RNA
HCC: Hepatocellular carcinoma
JHDM1D: Lysine demethylase 7A
NKG2D: Natural-killer group 2 member D
OS: Overall survival
qRT-PCR: quantitative real-time polymerase chain reaction
RFS: Recurrence-free survival
RIP: RNA immunoprecipitation
shRNA: small hairpin RNA
siRNAs: small interfering RNAs
TET: Ten eleven translocation
TP53INP1: Tumor protein p53 inducible nuclear protein 1
TRIM33: Tripartite motif containing 33
ULBP1: UL16 binding protein 1
WWC3: WWC family member 3
