# Supplementary material for: Circular RNA circTRIM33–12 acts as the sponge of MicroRNA-191 to suppress hepatocellular carcinoma progression
Source: Mol Cancer. 2019 Jun 1;18:105. doi: 10.1186/s12943-019-1031-1 (PMC6545035; doi:10.1186/s12943-019-1031-1)
Supplement: Supplementary file 1 — Supplementary Materials and Methods (DOC 34 kb) [file 12943_2019_1031_MOESM1_ESM.doc]

**Additional file 1: Supplementary Materials and Methods**

**Cell lines and clinical tissues**

MHCC97-L, MHCC97-H, HCCLM3 (LM3) (human HCC cell lines with different metastatic potentials were established at the Liver Cancer Institute of Zhong Shan Hospital, Fudan University, Shanghai, China); SMMC-7721 was obtained from the Institute of Biochemistry and Cell Biology at the Chinese Academy of Sciences (Shanghai, China), and the HepG2, Huh 7, Hep3B cell lines were purchased from the American Type Culture Collection (Manassas, VA). Briefly, all HCC cells were grown in Dulbecco’s Modified Eagle Medium (DMEM; Invitrogen, Grand Island, NY) supplemented with 10% fetal bovine serum (FBS; Invitrogen) and incubated at 37°C with 5% CO_2_ in a humidified atmosphere.

Clinical tissues taken from tumors and areas adjacent to the margin of tumors were collected from 150 consecutive patients with HCC who underwent curative resection between 2000 and 2002 at the Liver Cancer Institute of Fudan University (Shanghai, China). Parafﬁn blocks were selected only on the basis of the availability of suitable formalin-ﬁxed, parafﬁn-embedded tissue and complete clinicopathologic and follow-up data for the patients. The histopathological diagnosis was based on the World Health Organization (WHO) criteria. Ethical approval was obtained from the Zhongshan Hospital Research Ethics Committee, and written informed consent was obtained from each patient.

**Quantitative real-time polymerase chain reaction analysis, western blotting analysis, and immunofluorescence assays**

Gene amplification and detection were performed using the ABI PRISM 7900 Sequence Detection System (Applied Biosystems, Foster City, CA, USA) starting with 1 µl cDNA and SYBR Green Real-time PCR Master Mix (Takara, Japan). All transcript levels were normalized to glyceraldehyde 3-phosphate dehydrogenase (GAPDH) expression. Each sample was tested in triplicate. The relative expression was analyzed by the comparative cycle threshold (Ct) method, according to the equation 2-ΔCt [ΔCt = Ct-Ct (GAPDH)]. The reaction products of semiquantitative PCR analysis were run on a 2% agarose gel for semiquantitative detection by autoradiography. All experiments were performed in triplicate.

For western blotting, the total protein extracts from cells were separated by sodium dodecyl sulfate-polyacrylamide gel electrophoresis (SDS-PAGE), transferred onto polyvinylidene difluoride membranes, and incubated with the corresponding antibodies. The membranes were developed using the enhanced chemiluminescence method (Pierce, Rockford, IL, USA).

For the immunofluorescence assay, the cells were permeabilized with 0.1% Triton X-100 for 15 min at room temperature, washed with PBS, and blocked with PBS containing 5% bovine serum albumin (BSA) for 1 h at room temperature. The cells were treated with an antibody overnight at 4°C. The cells were rinsed with PBS and then incubated with the secondary antibody for 1 h at room temperature. The slices were counterstained with diamidino phenylindole (DAPI) and examined using fluorescence microscopy (Leica Microsystems Imaging Solutions, Cambridge, UK).

**circRNAs in vivo precipitation**

circTRIM33-12-overexpressing Huh 7 cells were washed with ice-cold phosphate-buffered saline (PBS), fixed with 1% formaldehyde, lysed in 500 μl co-IP buffer, sonicated and centrifuged. The supernatant was then added to a probes-M280 streptavidin dynabeads (Invitrogen) mixture and was further incubated at 30°C for 12 h. After that, to reverse the formaldehyde crosslinking, the probes-dynabeads-circRNAs mixture was washed and incubated with 200 μl lysis buffer and proteinase K. Subsequently, the RNA was extracted from the mixture using TRIzol Reagent (Invitrogen).

**Cell Proliferation, migration, and Matrigel invasion assay**

The cell proliferation assay was performed using a CCK-8 Kit (Dojindo Laboratories, Rockville, MD). Three hundred cells were seeded into each well in a 96-well plate. The CCK-8 solution (10 µl) was added to 100 µl of culture media, and the optical density was measured at 450 nm. Three independent experiments were performed.

Cell migration and invasion were measured using a transwell migration assay and a Matrigel invasion assay. For the transwell migration assay, 5 x 10^4^ cells were suspended in 200 µl of DMEM without serum and placed in the cell culture insert (8 µm pore size; BD Falcon, San Jose, CA) of a companion plate (BD Falcon) with a prewarmed culture medium containing 10% fetal bovine serum in the well. The cells were incubated for 12 h at 37°C in 5% CO_2_ and were then fixed with 4% paraformaldehyde in PBS. For the Matrigel invasion assay, 1 x 10^5^ cells were suspended in 200 µl of DMEM without serum and were placed in the cell culture insert precoated with 1 µg/µl Matrigel (BD Biosciences, San Jose, CA). A prewarmed culture medium containing 10% fetal bovine serum was added to the well. The cells were incubated for 24 h at 37°C in 5% CO_2_ and were then fixed with 4% paraformaldehyde in PBS. The nonmigrated or invaded cells on the top of the membrane were gently removed with a cotton swab. Cell migration or invasion was determined by staining cells with 0.1% crystal violet (Sigma, St Louis, MO) and counting the cells under a light microscope (100x magnification) in eight randomly selected areas.

**In vivo tumor growth and metastasis assays**

The in vivo tumor growth and metastasis assays were performed using nude mice. Athymic nude mice were obtained from the Shanghai Institute of Material Medicine and were maintained in a pathogen-free environment. Animal care and experimental protocols were performed in accordance with the guidelines established by the Shanghai Medical Experimental Animal Care Commission. Ethical approval was obtained from the Zhongshan Hospital Research Ethics Committee. HCC cells were injected intrahepatically with a 27-gauge needle. At six weeks, six nude mice in each group were euthanized by anesthesia overdose and the visceral organs, including the lungs and livers, were collected. Tumor sizes were evaluated by the formula: Volume (mm^3^) = [width^2^ (mm^2^) × length (mm)]/2. Intrahepatic and pulmonary metastatic foci were examined by pathological identification.

**Transfection experiment**

The miR-191 mimics and negative control were purchased from Shanghai GeneChem Co., Ltd. The TET cDNA and shRNA plasmids were purchased from Shanghai Yeasen Co., Ltd. The transfection of the miR-191 mimics, negative control, and plasmids was performed with Lipofectamine 2000 (Invitrogen, Carlsbad, CA, USA) according to the manufacturer’s instructions.

**Correlations between the two groups assay**

A positive reaction for TET1/NKG2D was scored in four grade categories depending on the intensity of the staining, i.e., 0, 1, 2 and 3, and the percentage of TET1/NKG2D-positive cells was also scored in four groups: 0 (0%), 1 (1 to 33%), 2 (34 to 66%) and 3 (67 to 100%). In cases with discrepancies between the duplicated cores, the higher score of the two tissues was taken as the final score. The sum of the intensity and percentage scores was used as the final staining score. Then, the correlations between the two groups were detected by analysis of Pearson’s correlation coefficient.
