# Supplementary material for: Circular RNA circTRIM33–12 acts as the sponge of MicroRNA-191 to suppress hepatocellular carcinoma progression
Source: Mol Cancer. 2019 Jun 1;18:105. doi: 10.1186/s12943-019-1031-1 (PMC6545035; doi:10.1186/s12943-019-1031-1)
Supplement: Supplementary file 2 — Table S1. Sequence of primers for qRT-PCR. (DOCX 17 kb) [file 12943_2019_1031_MOESM2_ESM.docx]

Additional file 2: Table S1. Sequence of primers for qRT-PCR.

| **Gene** | **Forward primer (5’-3’)** | **Reverse primer(5’-3’)** |
| --- | --- | --- |
| CircTRIM33-1 | CACACCTCCAAAGACAACTGC | GCAGGAGATGAAGCAGCCTG |
| CircTRIM33-2 | GAACACAAAGAACATAGTTGG | AAGCCAACTGCACTTGCATTG |
| CircTRIM33-3 | CACACCTCCAAAGACAAGATA | GCTGTAGTAACTTCATCTGTC |
| CircTRIM33-4 | TATACAGCAAGCGACTGAATG | GTAGTGCTGTGCTGCTGCCAC |
| CircTRIM33-5 | AGATGTTACAACAACAGCTGC | CACCAGCACCGCGGTGAGAG |
| CircTRIM33-6 | ATATTCCACCCATACAGTTGG | TCTTACATAGCCACTCTCCAC |
| CircTRIM33-7 | ACTGGCAGTCGAGGCAGTTGG | TCACAGGTGTGTGAGAATTGG |
| CircTRIM33-8 | GTGAAAGGTTTAATGAACACT | TTCTGGATTGGTAACTGAGGG |
| CircTRIM33-9 | GCAGAATGACATCACAGTTGG | AAGCCAACTGCACTTGCATTG |
| CircTRIM33-10 | GAACACAAAGAACATAGAGTC | CAATAGCTGACAGTCTCTACA |
| CircTRIM33-11 | GAATGTAGTCAATTTAGTTGG | CTACACAAAAGCCAACTGCAC |
| CircTRIM33-12 | GTGAAAGGTTTAATGAATTGG | CTCACAGGTGTGTGAGAATTG |
| CircTRIM33-13 | GGAGGAGTGCTTGCATGCACT | AGGGGATTAGCTCTATTGCTG |
| CircTRIM33-14 | GATATTCCACCCATACAGTAT | TGCTCCAACAGACTCTGAGAC |
| CircTRIM33-15 | ACTCAGGTGCAGAATAGTTGG | CTACACAAAAGCCAACTGCAC |
| CircTRIM33-16 | GAACACAAAGAACATAGGTAT | TGCTCCAACAGACTCTGAGAC |
| CircTRIM33-17 | ATATTCCACCCATACAGGTAT | TTCTACTCGTTTGTTAGTCTC |
| CircTRIM33-18 | CTACTTAGCTTTCCAAGTTGG | CTCACAGGTGTGTGAGAATTG |
| TET1 | GAGCAGCACGCATGAATTTGG | GTCTTGCATTGGAACCGAATC |
| WWC3 | AACAGTGGAGGCGAGAGCAAG | GAATATCCAGAGAATGAGCTG |
| TP53INP1 | GAAGAGTCACCTACTGAGCAC | ATAGACAGACATGCTGGGATG |
| ULBP1 | CTGAACCACAGTGGTGTGAAG | GGCTTCATGCTCACAAGACATC |
| JHDM1D | ACGGCAGCTGTGTTGGAGTAG | GCACTTGGGAAGACTCGAGAG |
| GAPDH | GGGGCTCTCCAGAACATCATCC | ACGCCTGCTTCACCACCTCTT |

**Abbreviations:** qRT-PCR, quantitative real-time polymerase chain reaction.
