# Supplementary material for: Circular RNA circTRIM33–12 acts as the sponge of MicroRNA-191 to suppress hepatocellular carcinoma progression
Source: Mol Cancer. 2019 Jun 1;18:105. doi: 10.1186/s12943-019-1031-1 (PMC6545035; doi:10.1186/s12943-019-1031-1)
Supplement: Supplementary file 3 — Table S2. Antibody for western blotting, immunofluorescence, RIP, and immunohistochemistry. (DOCX 17 kb) [file 12943_2019_1031_MOESM3_ESM.docx]

Additional file 3: Table S2. Antibody for western blotting, immunofluorescence, RIP, and immunohistochemistry.

| **Antibody** | **Company** | **Cat No.** |
| --- | --- | --- |
| 5mC | Abcam | ab10805 |
| 5hmC | Abcam | ab231902 |
| TET1 | Abcam | ab191698 |
| WWC3 | Abcam | ab108146 |
| TP53INP1 | Abcam | ab202026 |
| ULBP1 | Abcam | ab176566 |
| JHDM1D | Invitrogen | PA5-25040 |
| β-actin | Abcam | ab8226 |
| NKG2D | Abcam | ab203353 |
| HRP-labeled Goat Anti-Rabbit IgG(H+L) | Beyotime | A0208 |
| HRP-labeled Goat Anti-mouse IgG(H+L) | Beyotime | A0216 |
| Alexa Fluor 647-labeled Goat Anti-Mouse IgG(H+L) | Beyotime | A0473 |
| Alexa Fluor 488-labeled Goat Anti-Rabbit IgG(H+L) | Beyotime | A0423 |
| AGO2 | Abcam | ab32381 |
| IgG | Abcam | ab172730 |
