# Supplementary material for: Circular RNA circTRIM33–12 acts as the sponge of MicroRNA-191 to suppress hepatocellular carcinoma progression
Source: Mol Cancer. 2019 Jun 1;18:105. doi: 10.1186/s12943-019-1031-1 (PMC6545035; doi:10.1186/s12943-019-1031-1)
Supplement: Supplementary file 4 — Table S3. Target sequences of TET1 shRNA. (DOCX 15 kb) [file 12943_2019_1031_MOESM4_ESM.docx]

Additional file 4: Table S3. Target sequences of TET1 shRNA.

| **shRNA** | **Target sequence** |
| --- | --- |
| TET1 shRNA -1 | GATACTCCCTGCTTTGGGA |
| TET1 shRNA -2 | GGGACACGCTGTGGTGAAG |
| TET1 shRNA -3 | GCTACTGCAAATCAACAGG |
