# Supplementary material for: Circular RNA circTRIM33–12 acts as the sponge of MicroRNA-191 to suppress hepatocellular carcinoma progression
Source: Mol Cancer. 2019 Jun 1;18:105. doi: 10.1186/s12943-019-1031-1 (PMC6545035; doi:10.1186/s12943-019-1031-1)
Supplement: Supplementary file 5 — Table S4. circTRIM33–12 circRIP probe sequence. (DOCX 16 kb) [file 12943_2019_1031_MOESM5_ESM.docx]

Additional file 5: Table S4. circTRIM33-12 circRIP probe sequence.

| Name | Sequence |
| --- | --- |
| circTRIM33-12 circRIP probe | CTGAGATGTATCTACTTAGTAGATTATCTAAACTACTTGAGCCAG |
