# Supplementary material for: Circular RNA circTRIM33–12 acts as the sponge of MicroRNA-191 to suppress hepatocellular carcinoma progression
Source: Mol Cancer. 2019 Jun 1;18:105. doi: 10.1186/s12943-019-1031-1 (PMC6545035; doi:10.1186/s12943-019-1031-1)
Supplement: Supplementary file 6 — Figure S1. circTRIM33–12 expression in HCC cells. Figure S2. circTRIM33–12 regulated the progression of HCC cells in vitro. Figure S3. TET1 binds to miR-191 in HCC cells. Figure S4. Forced or reduced TET1 expression in HCC cells. Figure S5. TET1 regulated the progression of HCC cells in vitro. Figure S6. circTRIM33–12 and TET1 regulate the expression of several same genes in HCC cells. (DOCX 3576 kb) [file 12943_2019_1031_MOESM6_ESM.docx]

**Additional file 6: Figure and Figure Legends**

**
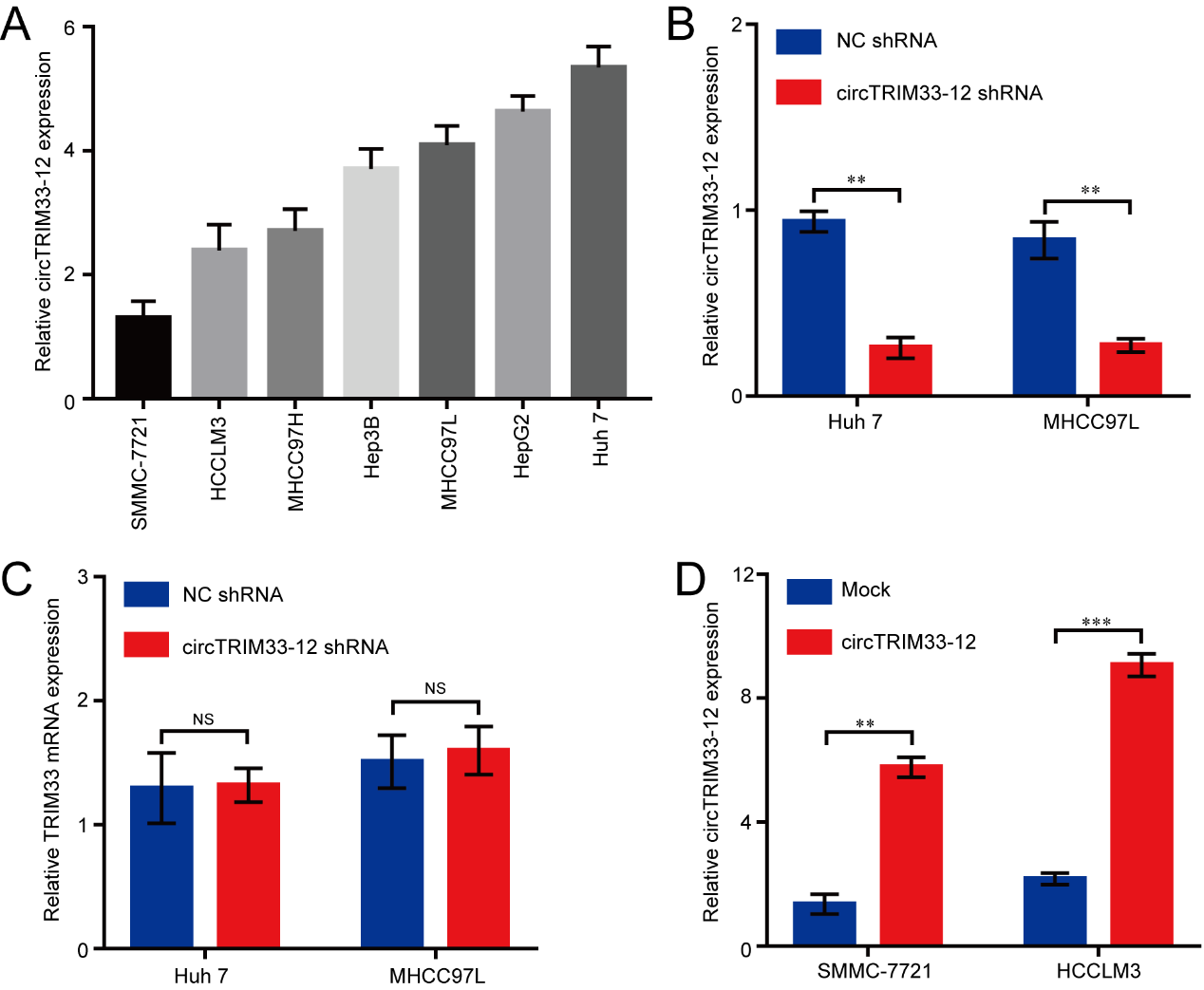
**

**Figure S1. circTRIM33-12 expression in HCC cells.** a circTRIM33-12 expression in several HCC cell lines was examined using qRT-PCR analysis. **b** circTRIM33-12 expression in Huh 7 cells was modified by shRNA interference transfection. **c** TRIM33-12 expression in circTRIM33-12 knockdown HCC cells. **d** circTRIM33-12 expression in Huh 7 cells was modified by cDNA transfection. The data are represented as the mean ± SD, n=3. **P < 0.01; ***P < 0.001; NS, no significant.


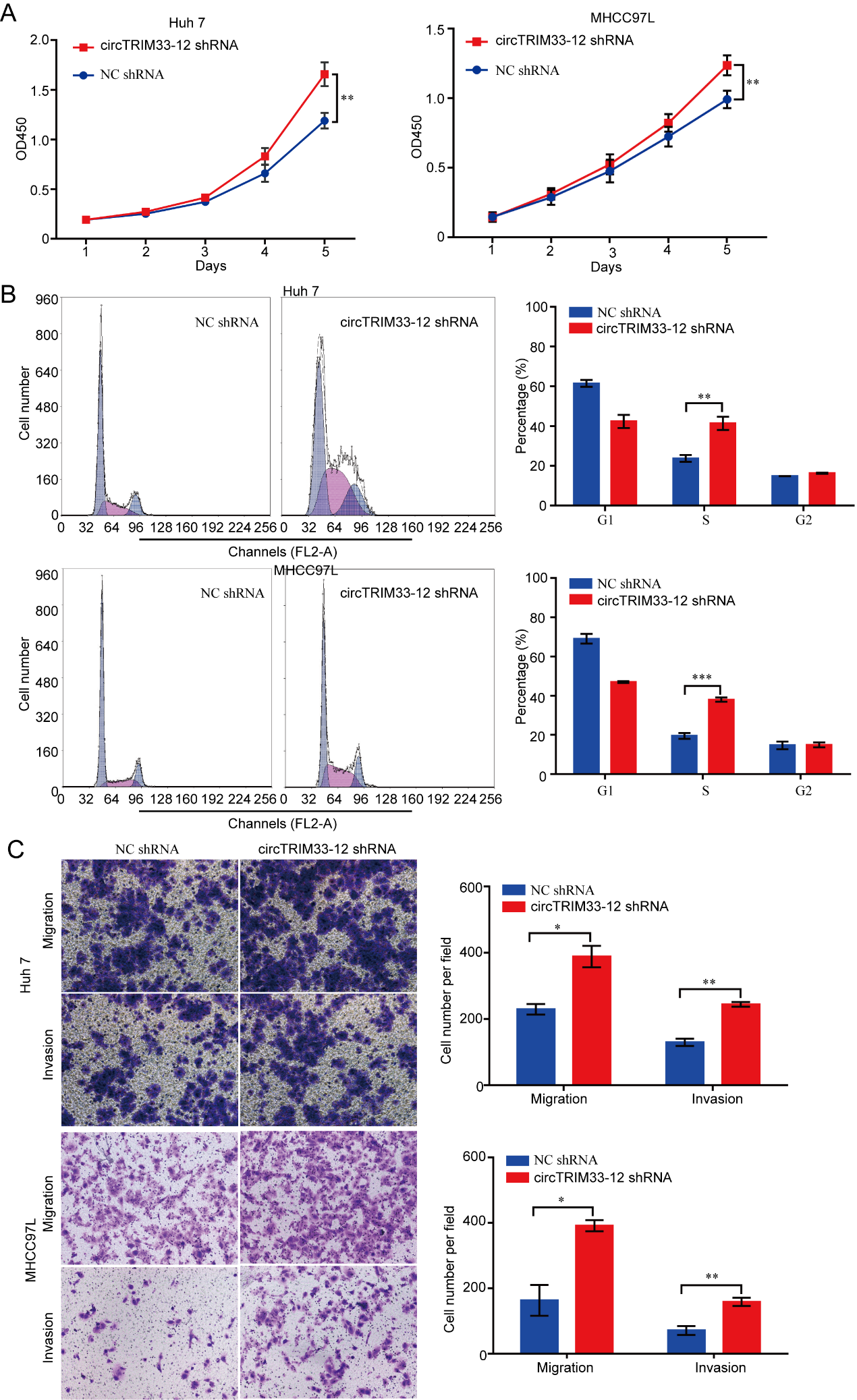


**Figure S2. circTRIM33-12 regulated the progression of HCC cells in vitro. a** Cell proliferation in HCC cells with the reduced expression of circTRIM33-12 was assessed by a CCK-8 assay. **b** The cell cycle in HCC cells with the reduced expression of circTRIM33-12 was detected by FCM. **c** The migration and invasion abilities in HCC cells with the reduced expression of circTRIM33-12 was evaluated via a transwell assay. The data are represented as the mean ± SD, n=3. *P < 0.05; **P < 0.01.


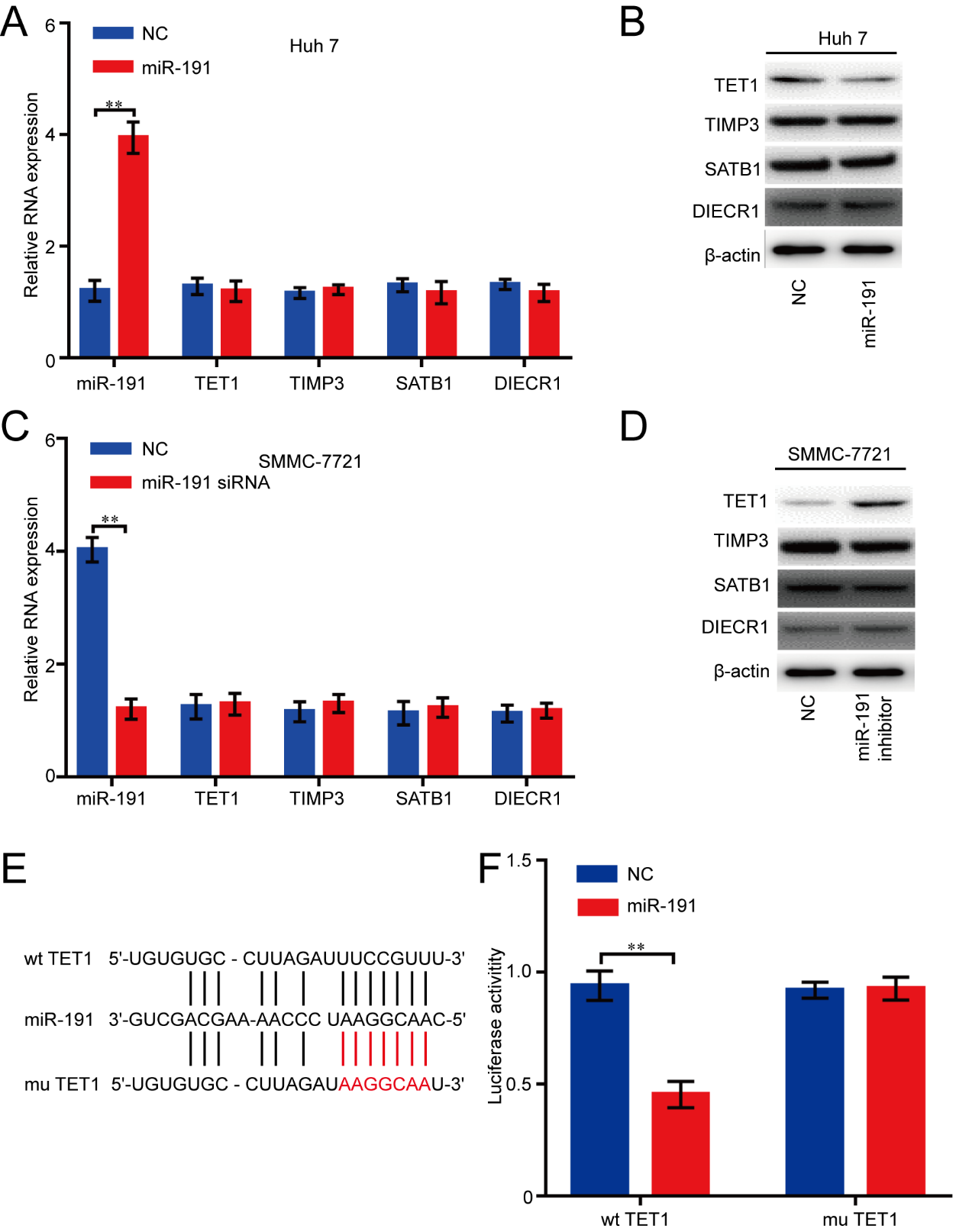


**Figure S3. TET1 binds to miR-191 in HCC cells. a** TET1, TIMP3, SATB1, and DIECR1 mRNA expression in Huh 7 cells was modified by miR-191 mimic transfection. **b** TET1, TIMP3, SATB1, and DIECR1 protein expression in Huh 7 cells was modified by miR-191 mimic transfection. **c** TET1, TIMP3, SATB1, and DIECR1 mRNA expression in SMMC-7721 cells was modified by miR-191 siRNA transfection. **d** TET1, TIMP3, SATB1, and DIECR1 protein expression in SMMC-7721 cells was modified by miR-191 siRNA transfection. **e** A schematic drawing showing the putative binding site of miR-191 with respect to TET1. **f** The luciferase activity of luc-TET1 or mutant luc-TET1 in SMMC-7721 cells after cotransfection with miR-191 or the negative control (NC). The data are represented as the mean ± SD, n=3. **P < 0.01.


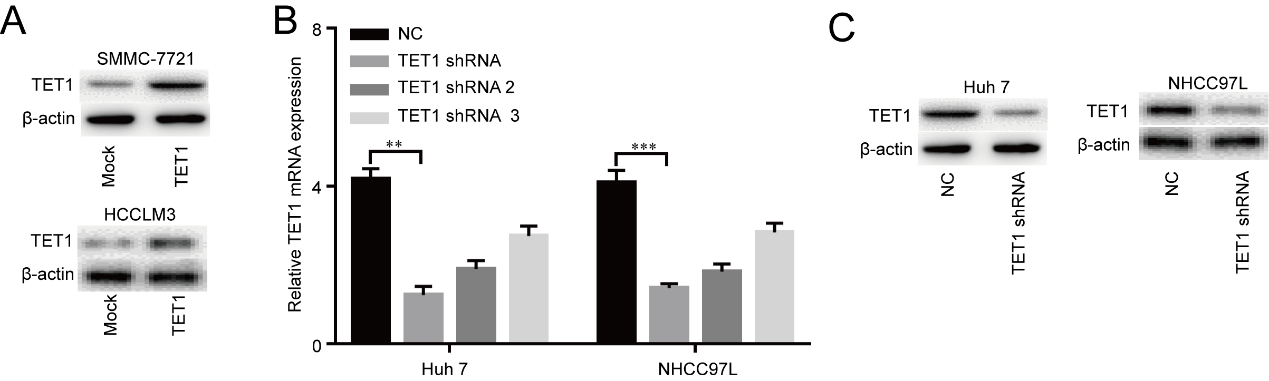


**Figure S4. Forced or reduced TET1 expression in HCC cells. a** TET1 expression in SMMC-7721 cells was modified by cDNA transfection. **b** TET1 mRNA expression in Huh 7 cells was modified by shRNA transfection. **c** TET1 protein expression in Huh 7 cells was modified by shRNA transfection. The data are represented as the mean ± SD, n=3. **P < 0.01; ***P < 0.001.


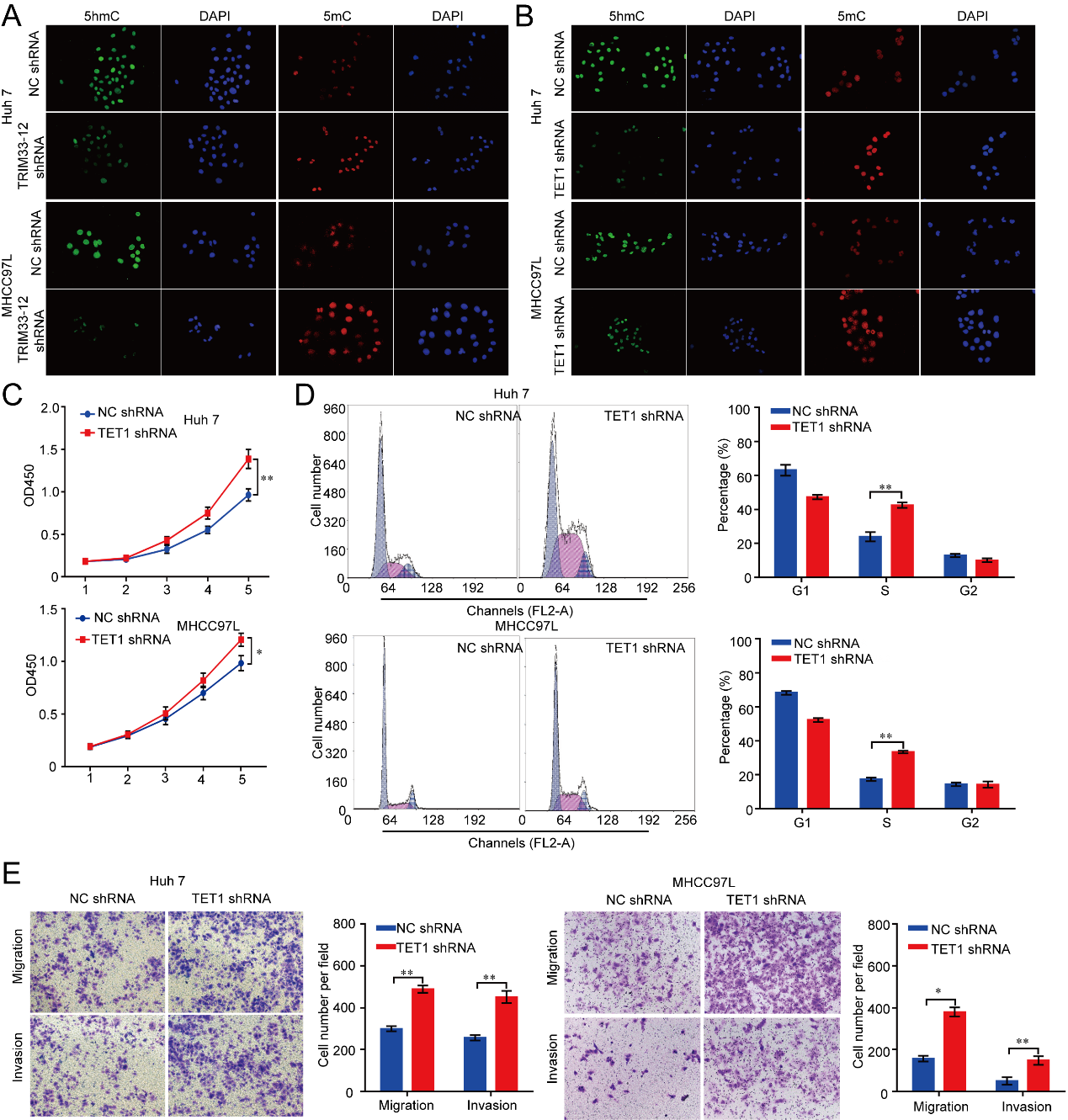


**Figure S5. TET1 regulated the progression of HCC cells in vitro. a** The expression of 5hmC and 5mC was detected in Huh 7 cells after transfection with circTRIM33-12 shRNA or the negative control (NC) using IF. **b** The expression of 5hmC and 5mC was detected in Huh 7 cells after transfection with TET1 shRNA or the negative control (NC) using IF. **c** Cell proliferation in Huh 7 cells with the reduced expression of TET1 was assessed by a CCK-8 assay. **d** The cell cycle in Huh 7 cells with the reduced expression of TET1 was detected by FCM. **e** The migration and invasion abilities of Huh 7 cells with the reduced expression of TET1 was evaluated via a transwell assay. The data are represented as the mean ± SD, n=3. *P < 0.05; **P < 0.01.


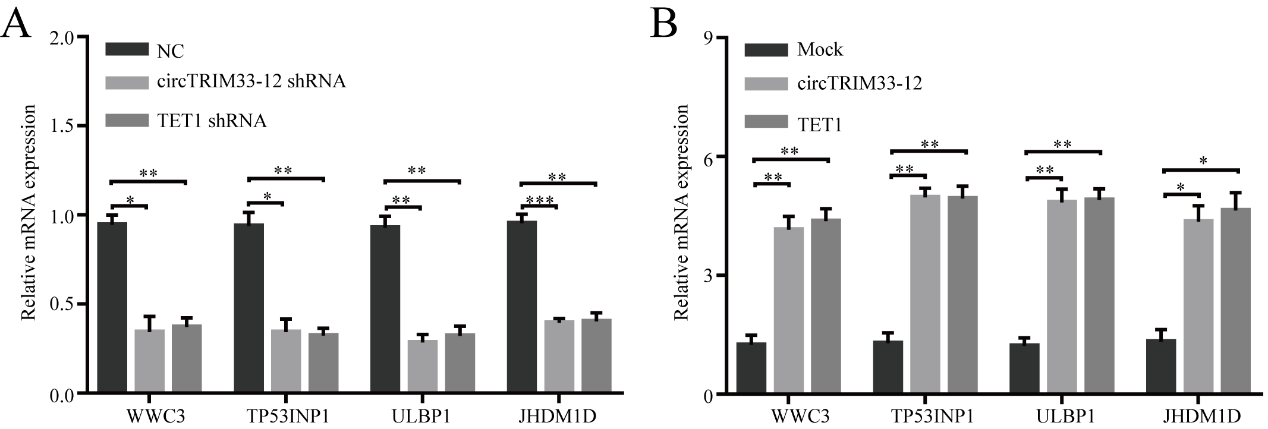


**Figure S6. circTRIM33-12 and TET1 regulate the expression of several same genes in HCC cells. a** The mRNA levels of WWC3, TP53INP1, ULBP1 and JHDM1D were detected in SMMC-7721 cells after transfection with circTRIM33-12, TET1, or the control using qRT-PCR. **b** The mRNA levels of WWC3, TP53INP1, ULBP1 and JHDM1D were detected in Huh 7 cells after transfection with circTRIM33-12 shRNA, TET1 shRNA, or the control using qRT-PCR. The data are represented as the mean ± SD, n=3. *P < 0.05; **P < 0.01; ***P < 0.001.
